# Assessing potency and binding kinetics of soluble adenylyl cyclase (sAC) inhibitors to maximize therapeutic potential

**DOI:** 10.3389/fphys.2022.1013845

**Published:** 2022-09-28

**Authors:** Thomas Rossetti, Jacob Ferreira, Lubna Ghanem, Hannes Buck, Clemens Steegborn, Robert W. Myers, Peter T. Meinke, Lonny R. Levin, Jochen Buck

**Affiliations:** ^1^ Department of Pharmacology, Weill Cornell Medicine, New York, NY, United States; ^2^ Department of Biochemistry, University of Bayreuth, Bayreuth, Germany; ^3^ Tri-Institutional Therapeutics Discovery Institute, New York, NY, United States

**Keywords:** soluble adenylyl cyclase, male contraceptive, residence time, drug development, picomolar potency, binding kinetics, lead optimization, SPR

## Abstract

In mammalian cells, 10 different adenylyl cyclases produce the ubiquitous second messenger, cyclic adenosine monophosphate (cAMP). Amongst these cAMP-generating enzymes, bicarbonate (HCO_3_^−^)-regulated soluble adenylyl cyclase (sAC; ADCY10) is uniquely essential in sperm for reproduction. For this reason, sAC has been proposed as a potential therapeutic target for non-hormonal contraceptives for men. Here, we describe key sAC-focused *in vitro* assays to identify and characterize sAC inhibitors for therapeutic use. The affinity and binding kinetics of an inhibitor can greatly influence *in vivo* efficacy, therefore, we developed improved assays for assessing these efficacy defining features.

## Introduction

Adenylyl cyclases (ACs) are the enzymes responsible for cyclizing adenosine triphosphate (ATP) into cyclic adenosine monophosphate (cAMP), a ubiquitous second messenger that plays an essential role in a wide variety of cellular signaling pathways. For many years following its initial discovery (Sutherland and Rall, 1958), it was widely thought that cAMP was exclusively produced at the plasma membrane of mammalian cells by nine different G-protein regulated, transmembrane-domain containing ACs (tmACs; *ADCY1-9*). However, in 1975, seemingly in opposition to this paradigm, a G-protein insensitive cytosolic AC activity was identified in a mammalian testis lysate (Braun and Dods, 1975). This cytosolic AC activity could utilize Mn^2+^ ions as a cofactor for the cyclization reaction, biochemically differentiating it from other class III adenylyl cyclases (like tmACs), which primarily relied on Mg^2+^ ions for AC activity (Braun and Dods, 1975; Braun et al., 1977). Together, these unique features indicated that this cytosolic AC activity was distinct from classical tmACs. Over 20 years later, a 10th mammalian AC gene (*ADCY10*) was cloned and its protein product, soluble AC (sAC), was identified as the enzyme responsible for the cytosolic AC activity (Buck et al., 1999). sAC is now recognized as one of 10 mammalian AC isoforms.

While sAC is the only mammalian AC isoform that lacks transmembrane spanning segments (Kamenetsky et al., 2006), like the other mammalian ACs, its enzymatic activity is attributed to two heterologous catalytic domains (C1 and C2) (Buck et al., 1999; Chen et al., 2000). RNA expression profiling studies predict the existence of sAC isoforms containing only the second (C2) catalytic domain (Farrell et al., 2008; Chen et al., 2013), thus far, only C1 and C2 containing isoforms of sAC have been biochemically characterized and demonstrated to be abundantly expressed in testes and sperm (Buck et al., 1999; Jaiswal and Conti, 2001; Hess et al., 2005). Further studies are needed to confirm the expression of, and understand the contribution to sAC biology of, C2-only isoforms. The experiments in this study focus exclusively on the C1-C2 containing isoforms.

Biochemically, both Ca^2+^ and HCO_3_^−^ directly and synergistically stimulate the activity of sAC; Ca^2+^ lowers the substrate K_m_ of sAC, thus increasing its apparent affinity for ATP, while HCO_3_^−^ increases the V_max_ of the reaction, thereby enhancing the rate at which sAC generates product cAMP (Litvin et al., 2003; Steegborn et al., 2005; Kleinboelting et al., 2014). Through these physiological activators, sAC plays a role in various biological processes (Wiggins et al., 2018; Rossetti et al., 2021) including reproduction (Buffone et al., 2014). Prior to ejaculation, sperm stored in the epididymis are morphologically mature, yet dormant, and lack the ability to fertilize an egg (Yanagimachi, 1994). Upon ejaculation, sperm begin to swim and gain the “capacity” to fertilize as they transit through the female reproductive tract in a molecular process called capacitation (Visconti et al., 1998). Pharmacological (Hess et al., 2005; Ramos-Espiritu et al., 2016; Balbach et al., 2021) and genetic (Esposito et al., 2004; Hess et al., 2005; Xie et al., 2006; Akbari et al., 2019) evidence, in both mice and humans, indicate that sAC generated cAMP is an essential component of the signal transduction pathway that mediates sperm motility and capacitation. Due to the essential role of sAC in these processes, there is great interest in developing sAC inhibitors for use as novel, on-demand, non-hormonal male contraceptives (Balbach et al., 2020; Ferreira et al., 2022). Despite its widespread expression (Wiggins et al., 2018; Rossetti et al., 2021), there are numerous strategies to safely and effectively target sAC for contraception (recently reviewed in Ferreira et al., 2022).

To develop sAC inhibitors for therapeutic use, we employed structure-based drug design to improve the potency and properties of a well-characterized, small molecule sAC inhibitor, LRE1 (Fushimi et al., 2021). LRE1 was identified in a high-throughput screen (Ramos-Espiritu et al., 2016). It is an allosteric inhibitor that binds to sAC with micromolar potency (IC_50_ = 3.2 μM, Table 1) at the dimer interface of C1 and C2 and occupies the same site where the activator HCO_3_^−^ binds (Kleinboelting et al., 2014). To assist in our drug discovery efforts, we developed multiple sAC-focused assays to thoroughly characterize sAC inhibitors. These assays are described in detail below. Assays for assessing inhibitor selectivity for sAC over tmACs (Ramos-Espiritu et al., 2016; Balbach et al., 2021) and inhibitor potency in physiologically relevant systems (Ramos-Espiritu et al., 2016; Balbach et al., 2021) were included in our drug discovery program, but they are not discussed as this paper focuses specifically on the *in vitro* characterization of inhibitor potency and binding kinetics.

**TABLE 1:** *In Vitro* Biochemical and Cellular Potency of sAC Inhibitors.

	sAC inhibitor structure	Standard assay IC_50_ (nM)	Subnanomolar assay IC_50_ (nM)	Cellular (4–4) IC_50_ (nM)
**LRE1**	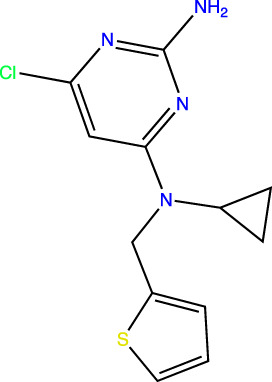	3,238	n/d	5,266
**TDI-10229**	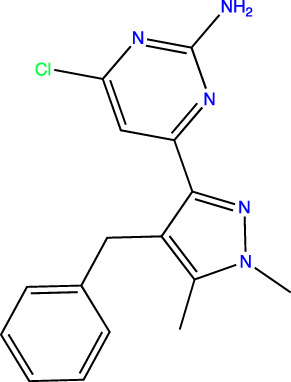	159	194	114
**TDI-11155**	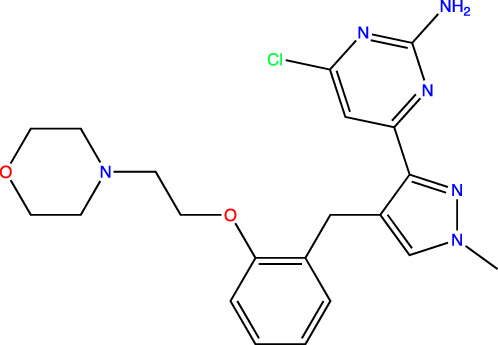	11	11	16
**TDI-11861**	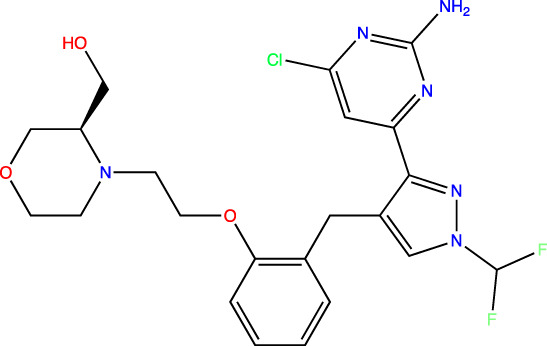	≤2.5	1.7	5
**TDI-11893**	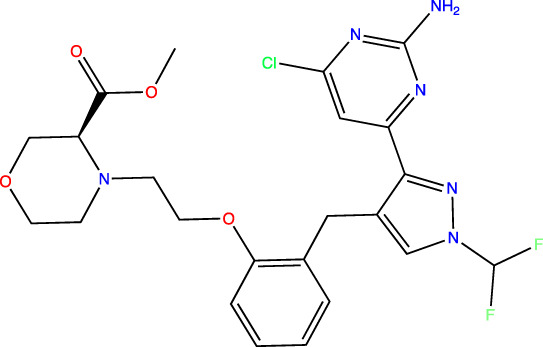	≤2.5	1.7	19
**TDI-11891**	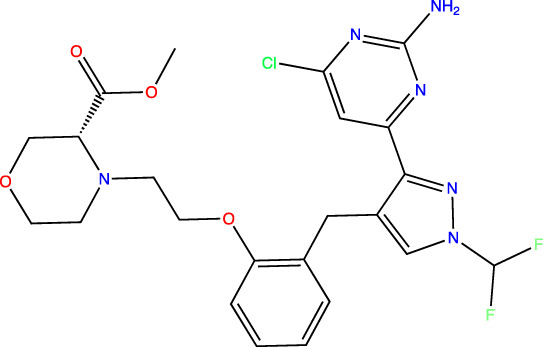	≤2.5	0.33	2.3

## Materials and methods

### Materials

Human sAC protein used in all assays was purified from Sf9 cells via baculovirus expression. Biochemical potency and jump dilution assays used N-terminal tagged GST-sAC_t_ (Litvin et al., 2003); SPR used highly pure C-terminal tagged sAC_t_-His6 that was prepared via sequential chromatography that included, in this order, affinity (Ni-Sepharose HP from GE Healthcare), ion exchange (Mono Q from GE Healthcare), and size exclusion (Superdex 200 from GE Healthcare) chromatographies. The synthesis of TDI-10229 is described in Fushimi et al., 2021 and the synthesis of other compounds is described in Miller et al., 2022.

### *In vitro* adenylyl cyclase activity assay with purified sAC protein (biochemical potency assay)

All *in vitro* adenylyl cyclase activity assays performed with purified human sAC protein utilized the classical “two-column” method, developed by Salomon (Salomon, 1979). In brief, conversion of α-^32^P labeled ATP into ^32^P labeled cAMP is quantitated following sequential Dowex and Alumina chromatography to purify the generated ^32^P labeled cAMP. Two variations of this assay were employed: the “standard” assay and the “subnanomolar” assay. In the standard assay, ∼5 nM of sAC_t_ protein was incubated in assay buffer containing 4 mM MgCl_2_, 2 mM CaCl_2_, 1 mM ATP, and 40 mM NaHCO_3_. The reaction was initiated by addition of ∼1,000,000 counts per minute of α-^32^P labeled ATP. In the subnanomolar assay, ∼0.25 nM of sAC_t_ protein was incubated in buffer containing 10 mM MnCl_2_ and 2 mM ATP, and the reaction was initiated by ∼3,000,000 counts per minute of *α*-^32^P labeled ATP Thus, this assay included three-fold higher specific activity of the substrate ATP. For both assay types, the buffer also contained 50 mM Tris-HCl pH 7.5, 3 mM DTT, and 0.03% BSA. Reactions were performed using a final volume of 100 μL at 30°C, and cAMP was quantitated after 30 min. For concentration-response curves, purified sAC_t_ protein was preincubated with the indicated inhibitors or vehicle (1% v/v DMSO) for 15 min. GraphPad Prism (www.graphpad.com, version 9.3.1) was used for curve fitting of the concentration-response data and determination of IC_50_ values.

### Cellular adenylyl cyclase activity assay in sAC overexpressing (4–4) cells (cellular potency assay)

To measure sAC-dependent cAMP accumulation in cells, we used human “4-4 cells”, which are HEK293 cells that stably overexpress sAC_t_ (Zippin et al., 2013). For the assay, 1 × 10^5^ 4-4 cells were seeded in each well of a 24-well plate and incubated for 24 h in DMEM+10% FBS media at 37°C/5% CO_2_. One hour before the assay the media was aspirated and replaced with 300 μL fresh media. Cells were preincubated with sAC inhibitor at the indicated concentrations or vehicle (0.7% DMSO) for 10 min at 37°C. Intracellular cAMP accumulation was initiated by addition of 500 μM IBMX (Sigma-Aldrich), and cAMP was allowed to accumulate for 5-min. Intracellular cAMP was quantitated using the Direct cAMP Elisa kit (Enzo Life Sciences) following manufacturer’s instructions. GraphPad Prism (www.graphpad.com, version 9.3.1) was used for curve fitting of the concentration-response data and determination of IC_50_ values.

### Surface plasmon resonance with purified sAC protein

On-rate (k_on_), off-rate (k_off_), and absolute affinity (K_i_) values for inhibitor binding to sAC were obtained at 25 or 37°C with a Biacore 8 K instrument (Cytiva) using a single cycle kinetics protocol. In PBS-P+ buffer (1 mM KH_2_PO_4_, 150 mM NaCl, 6 mM Na_2_HPO_4_, 0.05% (w/v) P20 Surfactant), 50 μg/ml of recombinant purified His-tagged sAC_t_ protein was covalently immobilized on a Series S Sensor NTA chip (Cytiva) using Ni^2+^-His tag chelation followed by amine coupling with a 1:1 mixture of 1-ethyl-3-(3-dimethylaminopropyl) carbodiimide and N-hydroxysuccinimide. After coupling, 1 M ethanolamine (Cytiva) followed by 350 mM EDTA (Cytiva) were, respectively, used to block any remaining reactive groups on the surface of the chip and to strip the Ni^2+^. Following chip preparation, TBS-P+ running buffer (50 mM Tris-HCl pH 7.5, 150 mM NaCl, and 0.05% (w/v) P20 Surfactant supplemented with 1% DMSO) was flowed over the surface of the chip. After a stable baseline was obtained, five different concentrations of the indicated inhibitors dissolved in TBS-P+ running buffer with a final DMSO concentration of 1%, were sequentially injected into a single channel for 120 s at a flowrate of 50 μl/min, followed by 60 s of TBS-P+ running buffer +1% DMSO alone. Concentrations used for TDI-10229 were 5,000, 1,250, 312.5, 78.1, and 19.5 nM. For all other inhibitors, the concentrations used were: 1,000, 250, 62.5, 15.6 and 4 nM. Subsequent to the highest concentration, compounds were allowed to dissociate for 600 s in the presence of TBS-P+ running buffer containing 1% DMSO. All experiments were performed in parallel in otherwise identically prepared reference channels lacking immobilized protein. To process the collected data, responses from the reference channels were subtracted from the responses from the active channels. From the reference-subtracted data, fitted curves, k_on_, k_off_, and K_i_ values were determined with the Biacore 8 K Insight Evaluation Software Version 2.0 (Cytiva) using a 1:1 binding kinetics model.

### *In vitro* jump dilution assay with purified sAC protein

Jump dilution adenylyl cyclase assays were performed using a modified version of the subnanomolar *in vitro* adenylyl cyclase activity assay described above. All assays were performed at 30°C, in the presence of 0.03% BSA, and each had a final DMSO concentration of 0.01%. At the start of each assay, ∼25 nM recombinant purified human sAC_t_ protein was preincubated with the indicated inhibitors for 15 min. Each inhibitor was used at an initial concentration 10-fold above their IC_50_ values. Following the preincubation period, 1 μl of the enzyme-inhibitor solution was diluted by addition of 99 μl of a reaction solution containing: 2 mM ATP, 10 mM Mn^2+^, 50 mM Tris pH 7.5, 3 mM DTT, 0.03% BSA and ∼4,000,000 counts per minute of α-^32^P labeled ATP. After this 100-fold dilution step, each inhibitor was present at a concentration 10-fold below their IC_50_ values to minimize inhibitor rebinding during the reaction. Parallel reactions were stopped every 6 min over the course of 1 h and generated cAMP was measured using the “two-column” method referenced above. Data were fit to the equation: % Total cAMP Formed = vst+((v_0_-v_s_) (1-e(^−^k_obs_^t^)))/k_obs_, with k_obs_ being an estimate of the dissociation rate constant (k_off_) (Kumar and Lowery, 2017) using GraphPad Prism (www.graphpad.com, version 9.3.1). To calculate v_s_ (uninhibited enzyme velocity) and v_0_ (inhibited enzyme velocity), the jump dilution assay was performed in the presence of only DMSO or an excess concentration of inhibitor, respectively.

## Results

### Assessing the biochemical and cellular potency of sAC inhibitors

The lead optimization phase of drug development is initially focused on efforts to improve the biochemical potency (IC_50_ for an inhibitor) of a lead compound via successive rounds of medicinal chemistry. Thus, a successful drug discovery program requires a robust potency assay that can efficiently and accurately determine the IC_50_ values of newly synthesized compounds. For our program, we utilized a highly reproducible, *in vitro* assay to determine the biochemical potencies of our sAC inhibitors on purified recombinant sAC protein. This *in vitro* biochemical potency assay utilized a radioactivity-based method to measure AC activity, which was pioneered by Yarom Salomon (Salomon, 1979). This method requires the use of α-^32^P labelled ATP that, in the presence of an active AC, is converted to ^32^P labelled cAMP. The radioactive cAMP reaction product is purified and separated from the unconverted radioactive ATP substrate using sequential Dowex and Alumina chromatography (Salomon et al., 1974). We routinely use this method to reliably measure the *in vitro* activity of several different ACs, including sAC (Levin et al., 1992; Chen et al., 2000; Litvin et al., 2003; Ramos-Espiritu et al., 2016).

**FIGURE 1:**
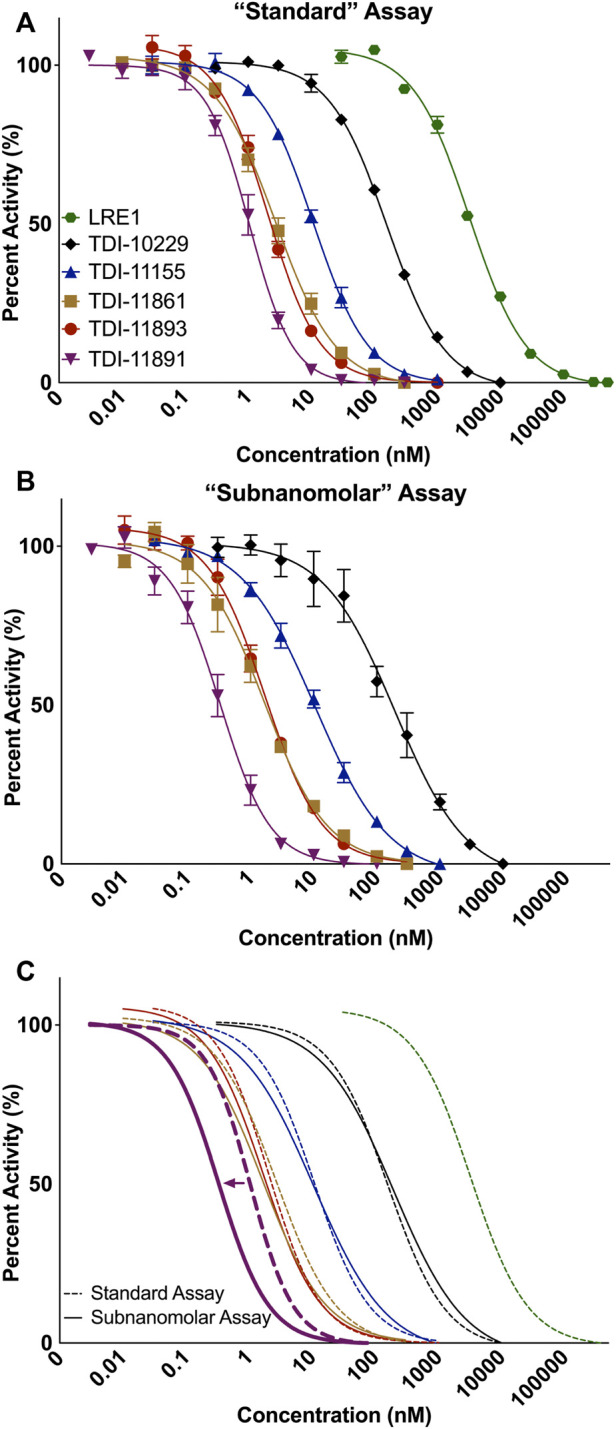
Measuring Biochemical IC_50_ Values with *In Vitro* sAC Activity Assays. Concentration-response curves of indicated inhibitors on purified recombinant human soluble adenylyl cyclase (sAC) protein⁠. Data was collected at 30°C in the presence of either **(A)** 1 mM ATP, 2 mM Ca^2+^, 4 mM Mg^2+^, 40 mM HCO_3_^−^ and ∼5 nM sAC protein or **(B)** 2 mM ATP, 10 mM Mn^2+^, and ∼0.25 nM sAC protein. Data is normalized to respective DMSO-treated controls and shown as mean ± SEM (*n* ≥ 3) **(C)** An overlay of the fitted IC_50_ curves from the standard (dashed lines) and subnanomolar (solid lines) assay. Colored arrow indicates the shift in IC_50_ for TDI-11891.

For our “standard” biochemical potency assay, the AC activity of ∼5 nM purified recombinant sAC protein is measured in a reaction that includes the physiologically relevant activators Ca^2+^ and HCO_3_^−^ (Litvin et al., 2003). For this assay, substrate ATP is kept at its K_m_ of 1 mM (Litvin et al., 2003), which closely mimics intracellular concentrations of ATP, to identify inhibitors that are either competitive, uncompetitive, or noncompetitive with substrate. Using our standard assay, we generated concentration-response curves for sAC inhibitors to calculate an accurate IC_50_ value via non-linear curve fitting (Figure 1A). Driven primarily by this standard assay to assess potency, we described the development and characterization of two sAC inhibitors, TDI-10229 (IC_50_ = 158.6 nM) (Balbach et al., 2021; Fushimi et al., 2021) and TDI-11861 (IC_50_ ≤ 2.5 nM) (Table 1) (Miller et al., 2022), that are useful for *in vivo* interrogation of sAC’s therapeutic potential.

In addition to TDI-11861, our medicinal chemistry efforts yielded numerous sAC inhibitors with biochemical potencies in the low nanomolar range (Miller et al., 2022) (Figure 1A; Table 1). When determining *in vitro* IC_50_ values, IC_50_ values of less than half the active target protein concentration cannot be measured due to the tight binding phenomenon (Williams and Morrison, 1979; Cook and Cleland, 2007). Since the concentration of sAC protein in our standard assay is ∼5 nM, the assay reached its theoretical limit and is not suitable for assessing inhibitor potencies of TDI-11861, TDI-11891, and TDI-11893, whose calculated IC_50_ values fell at or below 2.5 nM. To increase the potency range of our biochemical assay and gain the ability to measure low pM IC_50_ values, we tested lower concentrations of sAC protein in our cyclase assay (Supplementary Figure S1). We found 0.25 nM sAC produced sufficient cAMP over 30 min to assess inhibitor potencies. Thus, our new conditions decreased the amount of sAC protein 20-fold, from ∼5 to ∼0.25 nM. Because Mn^2+^-dependent *in vitro* sAC activity is ∼20-fold higher than Mg^2+^-dependent *in vitro* sAC activity (Litvin et al., 2003), we measured sAC activity in the presence of Mn^2+^ as the sole divalent cation. sAC activity was first detected in the presence of Mn^2+^ (Braun and Dods, 1975), and Mn^2+^ remains the most potent *in vitro* stimulator of sAC activity. TDI-10229 (Fushimi et al., 2021) and subsequent inhibitors (Miller et al., 2022) extend into the ATP binding site, so they inhibit both the physiologically-stimulated and Mn^2+^ stimulated activities. In parallel, we tripled the specific activity of the substrate ATP (i.e., increasing the radioactive ATP added to the assay from ∼1,000,000 to ∼3,000,000 counts per minute per assay). We refer to these assay conditions, with 20-fold reduced sAC protein levels, as a “subnanomolar” sAC activity assay.

We re-assessed potencies of several sAC inhibitors using this newly developed subnanomolar assay (Figure 1B). As expected, the inhibitors with IC_50_ values above 2.5 nM, TDI-10229 and TDI-11155, whose potencies are within the measurable range of the standard assay, had similar IC_50_ values in both assays (Figure 1C; Table 1). For the three sAC inhibitors with IC_50_ values at or near the theoretical lower limit of the standard assay (i.e., TDI-11861, TDI-11891, and TDI-11893), the subnanomolar assay revealed enhanced potencies. While TDI-11861 and TDI-11893 each exhibited IC_50_ values of 1.7 nM, TDI-11891, which is the enantiomer of TDI-11893 (Table 1), was ∼7.5-fold more potent with an IC_50_ of 0.33 nM in the subnanomolar assay (Figure 1C; Table 1). Thus, while the standard assay remains relevant for assessing potencies in the presence of the physiological activators Ca^2+^ and HCO_3_^−^, the newly developed assay conditions are suitable for distinguishing sAC inhibitors with subnanomolar potencies.

**FIGURE 2:**
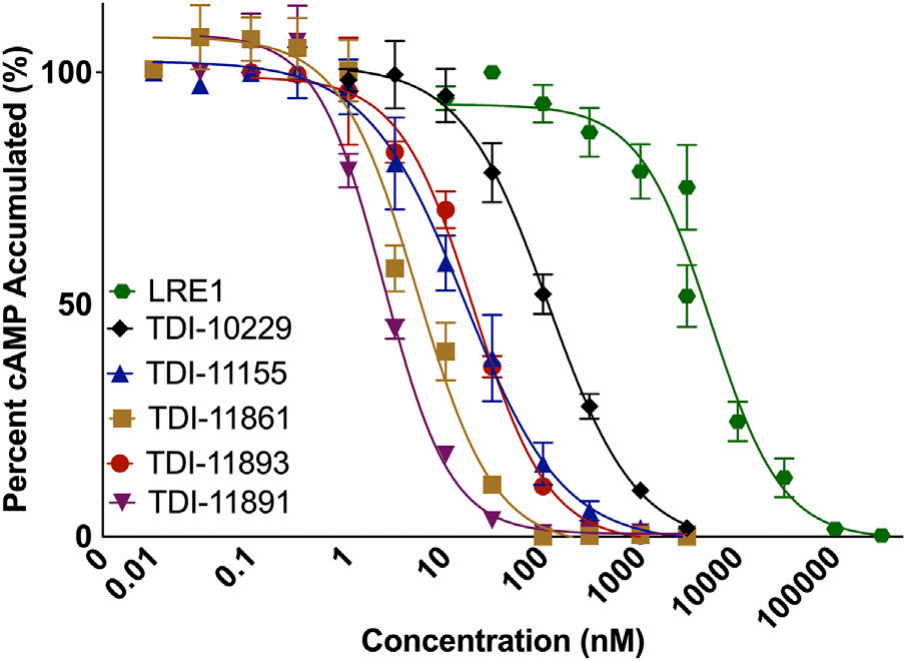
Measuring Cellular IC_50_ Values with a Cellular sAC Activity Assay. Concentration-response curves of indicated inhibitors in sAC-overexpressing 4-4 cells. Cells were preincubated with sAC inhibitors then treated with 500 μM 3-isobutyl-1-methylxanthine (IBMX) for 5 min. The amount of accumulated cAMP at 37°C was then measured. Data is normalized to respective DMSO-treated controls and shown as mean ± SEM (*n* ≥ 3).

While the standard and subnanomolar assays are useful tools for determining the potency of our inhibitors against purified sAC protein (i.e., biochemical potency), we are ultimately interested in inhibitor efficacy in a cellular context. In addition to how tightly an inhibitor binds its target, efficacy against an intracellular enzyme target is also determined by factors that are present only in the context of cells, such as membrane permeability, non-specific protein binding and compound stability in media and inside cells. To determine the cellular potency of our inhibitors, we developed a cell-based assay using engineered HEK293 cells that stably overexpress sAC, referred to as 4-4 cells (Zippin et al., 2013). sAC is the predominant source of cAMP inside 4-4 cells, and in the presence of a pan-selective phosphodiesterase (PDE) inhibitor (i.e., IBMX) that prevents cAMP degradation, cAMP accumulates within 4-4 cells in a sAC-dependent manner (Bitterman et al., 2013). Measuring cAMP accumulation in the presence of increasing doses of sAC inhibitors reveals their cellular potency. sAC inhibitors such as LRE1 (IC_50_ = 5.3 μM), TDI-10229 (IC_50_ = 113.5 nM), TDI-11155 (IC_50_ = 15.7 nM), and TDI-11861 (IC_50_ = 5.1 nM) had similar biochemical and cellular IC_50_ values while other sAC inhibitors, such as TDI-11893 (IC_50_ = 19.4 nM) and TDI-11891 (IC_50_ = 2.3 nM), had shifted cellular IC_50_ values compared to their biochemical IC_50_ values (Figure 2; Table 1), indicating cell-context specific influences on their potencies.

### Using surface plasmon resonance to measure affinities and binding kinetics of sAC inhibitors

A major goal of lead optimization is to obtain highly potent compounds. As detailed above, we achieved this goal by using sAC-focused *in vitro* potency assays to identify sAC inhibitors that had picomolar biochemical potencies and favorable low nanomolar cellular potencies. We were also extremely interested in inhibitor binding kinetics, especially the inhibitor dissociation rates. As an example, for sAC inhibitors to exhibit maximal efficacy as male contraceptives, compounds which bind sAC in sperm in the man will have to remain engaged with their target post-ejaculation, after the sperm enter the inhibitor-free female reproductive tract.

The binding kinetics of an inhibitor involves the various rates that define the interactions between the inhibitor and its target protein; those being the bimolecular rate at which the inhibitor binds to its target (i.e., k_on_, association constant, or on-rate) and the rate at which the target-inhibitor complex dissociates (i.e., k_off_, dissociation constant, or off-rate). The on-rate and off-rate of an inhibitor define its absolute biochemical affinity, as K_i_ = k_off_/k_on_. Consequently, a high intrinsic affinity is an indication of either a slow off-rate, a fast on-rate, or both. Another useful metric that can be evaluated and enhanced during drug development is the residence time (*τ*), which is defined as the reciprocal of the off-rate (1/k_off_) and is a estimation of the total lifetime of the inhibitor-target complex (Copeland, 2016; Bernetti et al., 2019). It is well precedented that a significant percentage of drug candidates have strong preclinical data but ultimately still fail in clinical trials due to a lack of efficacy (Kola and Landis, 2004). Recent evidence from a variety of drug discovery efforts indicates that residence time can be a major determinant of *in vivo* efficacy, defining it as an important inhibitor property to be optimized during lead optimization (Copeland et al., 2006; Swinney, 2008; Lu and Tonge, 2010; Schuetz et al., 2017).

**FIGURE 3:**
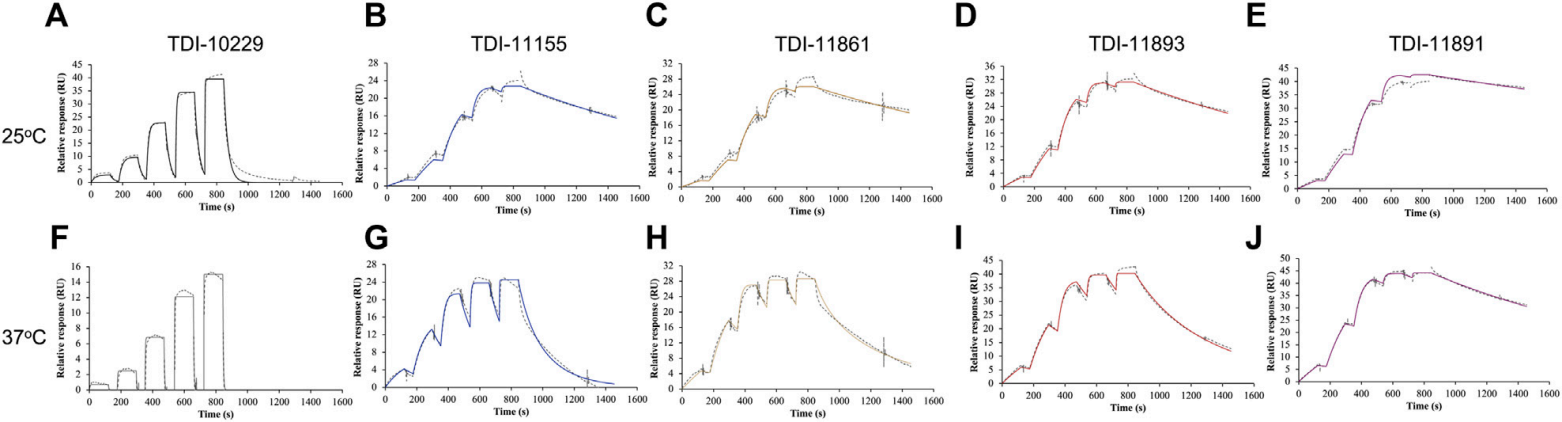
Assessing sAC Inhibitor Binding Kinetics with Surface Plasmon Resonance. Representative single-cycle SPR sensorgrams of **(A,F)** TDI-10229, **(B,G)** TDI-11155, **(C,H)** TDI-11861 **(D,I)** TDI-11893, and **(E,J)** TDI-11891 associating to and dissociating from immobilized sAC protein. Traces (dotted lines) are shown along with best fits generated via a 1:1 binding model (colored lines). Experiments (n ≥ 4) were conducted at either **(A–E)** 25°C or **(F–J)** 37°C.

To incorporate residence time assessment into our drug discovery program and define the absolute affinity of the inhibitors, we employed surface plasmon resonance (SPR), a biophysical technique that is widely utilized to measure both the on-rates and off-rates of biomolecular interactions (Zhang et al., 2016). For SPR, we sequentially flowed increasing concentrations of each sAC inhibitor over a sensor chip containing covalently immobilized sAC protein. Inhibitors were applied for 120 s to determine the on-rate (k_on_), followed by 60 s of buffer. After the final, highest concentration was bound, the sAC inhibitor was allowed to dissociate for 600 s to measure the off-rate (k_off_). The on-rate, off-rate, K_i_, and residence time values were calculated by evaluation software using a 1:1 binding kinetics model. Residence times were found to increase with the biochemical potency of the inhibitors (i.e., the more potent sAC inhibitors had longer residence times) (Figures 3A–E; Table 2). For example, at room temperature (25°C) a sAC inhibitor with mid-nanomolar biochemical potency (TDI-10229, IC_50_ = 158.6 nM) had the shortest residence time (25 s) of the compounds tested (Figure 3A), while a sAC inhibitor with a mid-picomolar biochemical potency (TDI-11891, biochemical IC_50_ = 0.33 nM) had a significantly longer residence time of ∼3,000 s (Figure 3E). Among the sAC inhibitors tested, the on-rates, which are lower than diffusion controlled, did not change with respect to biochemical potency; the assessed sAC inhibitors had on-rates of ∼200,000 (Ms)^−1^ regardless of their biochemical potency (Table 2). Thus, the observed correlation between measured absolute affinity (K_i_) (Table 2) and biochemical potencies (Table 1) reflects differences in inhibitor residence times.

**TABLE 2:** Binding Kinetics and Absolute Affinities of sAC Inhibitors.

	25°C SPR on-rate, k_on_ (x10^5^ (Ms)^−1^)	25°C SPR residence time, τ (sec)	25°C SPR affinity, K_i_ (nM)	37°C SPR on-rate, k_on_ (x10^5^ (Ms)^−1^)	37°C SPR residence time, τ (sec)	37°C SPR affinity, K_i_ (nM)	30°C jump dilution residence time, τ (sec)
TDI-10229	2.3 ± 0.3	25 ± 1	193 ± 27	3.71 ± 1.23	8 ± 2	423 ± 16	<1
TDI-11155	1.9 ± 0.2	1,474 ± 98	3.8 ± 0.4	5.49 ± 0.96	228 ± 34	8.8 ± 0.7	666 ± 33
TDI-11861	2.1 ± 0.2	2,219 ± 98	2.5 ± 0.3	4.90 ± 0.13	391 ± 81	4.5 ± 0.6	1,073 ± 32
TDI-11893	2.1 ± 0.2	1,672 ± 134	3.1 ± 0.3	4.79 ± 0.36	406 ± 38	5.3 ± 0.4	1,483 ± 73
TDI-11891	1.8 ± 0.3	3,103 ± 571	1.8 ± 0.4	3.30 ± 0.29	1,116 ± 270	3.7 ± 1.4	5,679 ± 234

These SPR experiments were performed at room temperature (25°C) which facilitates measuring off-rates of compounds with shorter residence times. For compounds with long residence times, we repeated the SPR experiments at the physiologically relevant temperature of 37°C. As expected, the elevated temperature speeds up the molecular interactions occurring within the system, leading to accelerated on-rates and shortened residence times. Increasing the temperature significantly reduced sAC inhibitor residence times by 3- to 6.5-fold, increased on-rates by 1.6- to 3-fold, and increased K_i_ values by approximately 2-fold (Figures 3F–J; Table 2). However, at 37°C, the measured residence times and absolute affinities still positively correlated with biochemical potency (Table 1) (i.e., the most potent sAC inhibitors exhibited the longest residence times and the strongest absolute affinities).

### An *in vitro* jump dilution assay to measure sAC inhibitor residence times

**FIGURE 4:**
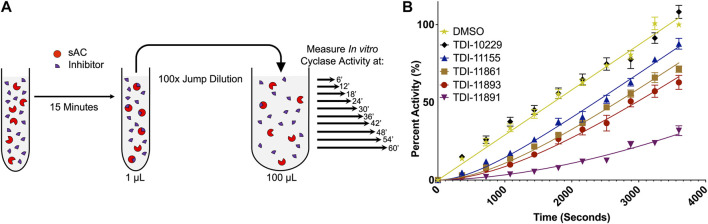
*In Vitro* Jump Dilution Assay for Determining sAC Inhibitor Residence Times. **(A)** Schematic diagram of the jump dilution assay (figure adapted from BellBrook Labs “A Guide to Measuring Drug Target Residence Times with Biochemical Assays”) **(B)** *In vitro* jump dilution curves of indicated inhibitors. All assays were done at 30°C in the presence of 2 mM ATP, 10 mM Mn^2+^ and ∼0.25 nM of purified recombinant human sAC protein. Following a 100-fold dilution, sAC cyclase activity was measured every 6 min for 60 min. Data is normalized to respective DMSO-treated controls and is shown as mean ± SEM (*n* ≥ 4).

SPR is a valuable technique to directly compare the binding kinetics and absolute affinities of sAC inhibitors, but it is a purely biophysical technique that studies the inhibitor/target interaction with the enzyme immobilized. Therefore, we also developed an *in vitro “*jump dilution” adenylyl cyclase assay. Jump dilution assays are an experimental format frequently used as an alternative/complement to SPR to measure the residence time of small molecules (Walkup et al., 2015; Zhang et al., 2016). Our *in vitro* jump dilution cyclase assay was a modified version of the subnanomolar biochemical potency assay. Each sAC inhibitor was preincubated with sAC protein at an inhibitor concentration 10-fold above its biochemical IC_50_ and a sAC concentration 100-fold above its level in the subnanomolar sAC activity assay. After a set preincubation time, the protein-inhibitor mixture underwent a 100-fold “jump dilution,” such that the final inhibitor concentration is 10-fold below its biochemical IC_50_ (Figure 4A). At this minimal inhibitory concentration, sAC activity recovered as the inhibitor dissociated from the protein over the course of the 60-min experiment (Figure 4B). The off-rate of each inhibitor is the rate at which sAC activity recovered to uninhibited levels; the reciprocal of this rate is the inhibitor residence time (Kumar and Lowery, 2017).

As expected, the rank order of residence times determined with the newly developed *in vitro* jump dilution assay correlated well with the order of compound residence times measured via SPR (Table 2). However, the exact length of the residence time differed in the two assays, and this difference, either an increase or decrease in residence time, varied from inhibitor to inhibitor. For example, TDI-11861 had a 2-fold shorter residence time in the jump dilution assay (∼2,200 s vs. ∼1,000 s) while TDI-11891 had a 2-fold longer residence time in the jump dilution assay (∼3,100 s vs. ∼5,700 s) (Table 2). The maximum length of our *in vitro* jump dilution assay is 60 min (3,600 s), and thus the overall length of the assay was shorter than the measured residence time of TDI-11891. Since the length of a jump dilution assay should exceed the longest measured residence time (Zhang et al., 2016), we increased the jump dilution assay runtime to 180 min for TDI-11891, and measured sAC activity every 18 min (Supplementary Figure S2A). In this extended jump dilution assay, the measured residence time of TDI-11891 increased only slightly to ∼6,500 s (Supplementary Figure S2B).

## Discussion

sAC generated cAMP-signaling has been linked to various physiological processes, including lysosomal acidification (Rahman et al., 2016), regulation of intraocular eye pressure (Lee et al., 2011; Gandhi et al., 2017), and the motility and capacitation of sperm (Hess et al., 2005; Xie et al., 2006; Buffone et al., 2014). Due to these varied biological functions, sAC inhibition is a novel approach with several potential therapeutic applications (Wiggins et al., 2018; Ferreira et al., 2022). To develop sAC inhibitors for therapeutic use, we initiated drug discovery efforts that sought to bring a compound identified in a high-throughput screen (LRE1) through the lead optimization phase to ultimately produce sAC inhibitors with “drug-like” properties. In this paper, we detail several sAC-focused *in vitro* assays that we used to thoroughly characterize efficacy defining properties of sAC inhibitors and, as a result, establish vital tools in sAC-directed drug discovery research.

For *in vitro* potency assessment, we use two assays; our previously described standard assay, which measures potency in a reaction containing physiologically relevant sAC activators (Balbach et al., 2021; Fushimi et al., 2021; Miller et al., 2022), and the newly developed subnanomolar assay, which is used to determine the biochemical potency of our highest affinity sAC inhibitors. The subnanomolar assay overcomes the protein concentration-mediated limitations of the standard assay, affording us the capability to reliably determine IC_50_ values for sAC inhibitors whose biochemical potencies are in the high-to mid-picomolar range. The new dynamic range achieved with the subnanomolar assay allows us to continue our structure-guided medicinal chemistry efforts to further improve the biochemical potencies of sAC inhibitors. However, the subnanomolar assay does have limitations because it includes Mn^2+^ as the sole divalent cation in the assay and does not include allosteric modulators, such as HCO_3_^−^. The compounds used in this study, in addition to allosterically inhibiting sAC via binding in the bicarbonate binding site (Ramos-Espiritu et al., 2016), interfere with ATP binding (Fushimi et al., 2021; Miller et al., 2022). To complement and expand the standard and subnanomolar sAC activity assays, we have also incorporated a cellular potency assay into our drug discovery program. We use this assay to identify sAC inhibitors that function in a cellular context, eliminating compounds with poor cellular target engagement from further development.

Our lead optimization program also utilizes two separate assays, SPR and *in vitro* jump dilution, to assess the binding kinetics (i.e., on-rate and/or off-rate) of our sAC inhibitors. We conduct our SPR experiments at two different temperatures, 25°C and the more physiologically relevant 37°C. As expected, sAC inhibitor residence times are much shorter at 37°C, providing a more realistic evaluation of functional residence times during *in vivo* experiments. For this chemical series of sAC inhibitors, the prime determinant for absolute affinity and biochemical potency appears to be residence time, which generally increased concurrently with both properties; on-rates of our sAC inhibitors did not change in relation to potency or affinity. To complement our SPR experiments, which were done in absence of any AC reaction cofactors and/or substrate on immobilized protein, we developed an *in vitro* jump dilution assay to determine if sAC inhibitor residence times were altered in the presence of an active, cAMP-producing enzyme. The residence times measured *via in vitro* jump dilution assays are consistent with the results from SPR experiments; the rank order of sAC inhibitor residence times is similar between the two types of assays. For some sAC inhibitors, we observe up to 2-fold differences between *in vitro* jump dilution residence times and SPR residence times, which could be due to different assay temperatures, inter-assay variability or altered inhibitor binding due to the presence of ATP/Mn^2+^ and the allosteric changes that occur during the enzymatic reaction. Finally, we increased the length of the *in vitro* jump dilution assay to reliably determine the residence time for the tightest binding sAC inhibitor, TDI-11891, whose residence time is longer than 3,600 s. Although lengthening the assay did not reveal an appreciable difference for TDI-11891, the extended jump dilution assay allows for a reliable assessment of inhibitors with significantly longer residence times. This may be required if, in the future, our drug discovery program generates sAC inhibitors with residence times even greater than TDI-11891.

The assays described in this paper are important research tools that can be utilized to characterize sAC inhibitors. They comprise the initial stages of a workflow designed to produce sAC inhibitors with optimized therapeutic properties (i.e., high potency and long residence times). The standard assay is used for initial assessment of the biochemical potency of a sAC inhibitor; if the measured IC_50_ value is less than or equal to the theoretical limit of the standard assay (∼2.5 nM) then it is followed by the subnanomolar assay to determine whether the true IC_50_ is lower than originally measured. Once sAC inhibitors with the desired *in vitro* potencies are identified, the cellular potency (4-4 cell) assay is used to exclude sAC inhibitors with cell-specific limitations from further assessment and development. In parallel, residence times and absolute affinity are determined via SPR at room temperature, and if sufficiently tight binding, at 37°C, to best mimic binding kinetics that will occur *in vivo*. The jump dilution assay is then used as an independent check to confirm the measured residence times and to determine whether the residence time may be altered due to allosteric changes during the enzymatic reaction. For inhibitors whose measured residence time exceeds 3,600 s, the extended jump dilution will reliably determine a significantly long residence time. At this point in the process, promising sAC inhibitors should be assessed for sAC-selectivity. The mammalian enzymes most evolutionarily and biochemically similar to sAC are the family of 9 transmembrane adenylyl cyclases (tmACs); thus, we examine inhibitory activity against several tmACs (Bitterman et al., 2013; Ramos-Espiritu et al., 2016; Fushimi et al., 2021). Potent and selective inhibitor candidates are then subjected to a pharmacodynamic efficacy assay. As mentioned previously, sAC is essential for sperm capacitation (Esposito et al., 2004; Hess et al., 2014; Akbari et al., 2019; Balbach et al., 2021), so for this application we measure concentration dependence in several sperm capacitation-specific assays (Ramos-Espiritu et al., 2016; Balbach et al., 2021). These assays are directly applicable if sAC inhibitors are being developed as contraceptives. If sAC inhibitors are being developed for another therapeutic use, then other function-specific assays need to be conducted to determine inhibitor potency in physiologically relevant systems. Ultimately, when the ideal candidate sAC inhibitor is identified, the *in vivo* efficacy of the inhibitor must be determined in an appropriate *in vivo* experiment to provide proof-of-concept evidence for the therapeutic benefit of sAC inhibition.

In summary, what we report here is a workflow designed for vetting sAC inhibitors for drug discovery programs where binding kinetics, specifically residence times, and affinity are efficacy defining features.

## Data Availability

The raw data supporting the conclusion of this article will be made available by the authors, without undue reservation.
