# Erratum: Assessing potency and binding kinetics of soluble adenylyl cyclase (sAC) inhibitors to maximize therapeutic potential

**DOI:** 10.3389/fphys.2022.1092217

**Published:** 2022-11-21

**Authors:** Frontiers Production Office

**Affiliations:** Frontiers Media SA, Lausanne, Switzerland

**Keywords:** soluble adenylyl cyclase, male contraceptive, residence time, drug development, picomolar potency, binding kinetics, lead optimization, SPR

Due to a production error, Figure 4 was a duplication of **Figure 3**. The correct Figure 4 is shown below

**FIGURE 4:**
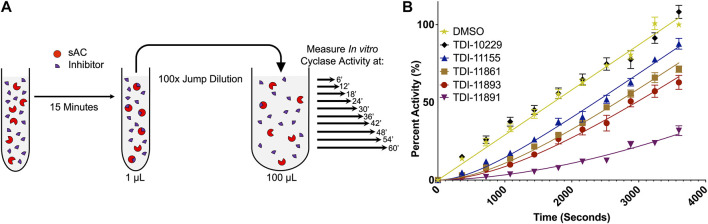
*In Vitro* Jump Dilution Assay for Determining sAC Inhibitor Residence Times. **(A)** Schematic diagram of the jump dilution assay (figure adapted from BellBrook Labs “A Guide to Measuring Drug Target Residence Times with Biochemical Assays”). **(B)** *In vitro* jump dilution curves of indicated inhibitors. All assays were done at 30°C in the presence of 2 mM ATP, 10 mM Mn^2+^ and ∼0.25 nM of purified recombinant human sAC protein. Following a 100-fold dilution, sAC cyclase activity was measured every 6 min for 60 min. Data is normalized to respective DMSO-treated controls and is shown as mean ± SEM (*n* ≥ 4).

The publisher apologizes for the mistake. The original article has been updated.

