# Corrigendum: Assessing potency and binding kinetics of soluble adenylyl cyclase (sAC) inhibitors to maximize therapeutic potential

**DOI:** 10.3389/fphys.2023.1163389

**Published:** 2023-03-06

**Authors:** Thomas Rossetti, Jacob Ferreira, Lubna Ghanem, Hannes Buck, Clemens Steegborn, Robert W. Myers, Peter T. Meinke, Lonny R. Levin, Jochen Buck

**Affiliations:** ^1^ Department of Pharmacology, Weill Cornell Medicine, New York, NY, United States; ^2^ Department of Biochemistry, University of Bayreuth, Bayreuth, Germany; ^3^ Tri-Institutional Therapeutics Discovery Institute, New York, NY, United States

**Keywords:** soluble adenylyl cyclase, male contraceptive, residence time, drug development, picomolar potency, binding kinetics, lead optimization, SPR

In the original article, there was an error in Table 1 as published. In this table, the chemical structures of TDI-11861 and TDI-11155 were incorrect as the chloro and amino groups of the pyrimidine were drawn in the incorrect positions. In the correct chemical structures, the chloro and amino groups are located at the 4- and 2-positions, respectively. The corrected Table 1 appears below.

**TABLE 1:** *In Vitro* Biochemical and Cellular Potency of sAC Inhibitors.

	sAC inhibitor structure	Standard assay IC_50_ (nM)	Subnanomolar assay IC_50_ (nM)	Cellular (4-4) IC_50_ (nM)
LRE1	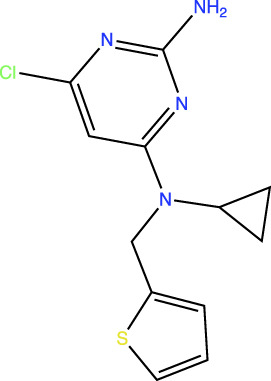	3238	n/d	5266
TDI-10229	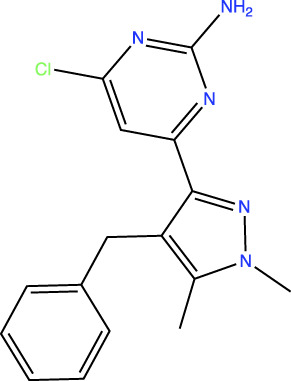	159	194	114
TDI-11155	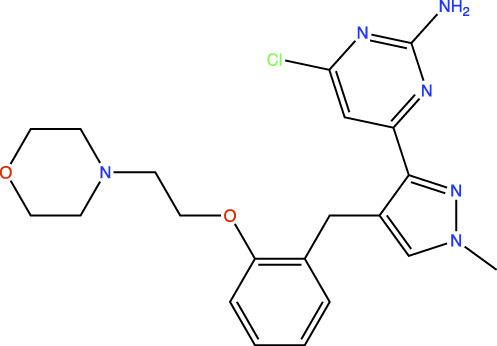	11	11	16
TDI-11861	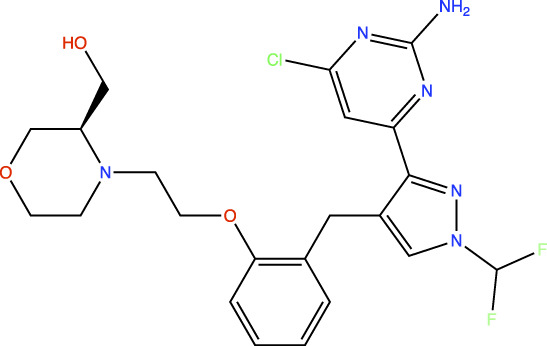	≤2.5	1.7	5
TDI-11893	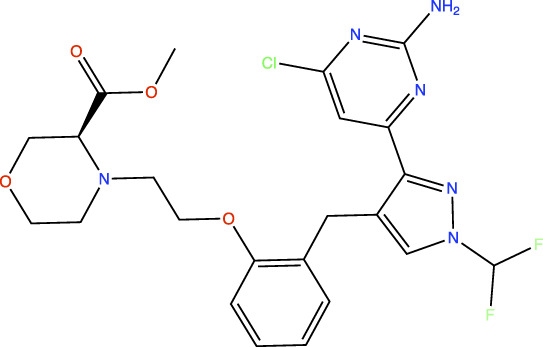	≤2.5	1.7	19
TDI-11891	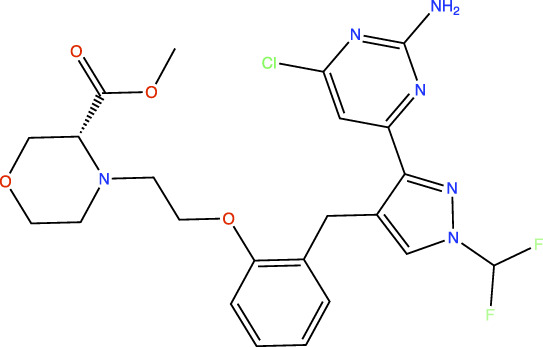	≤2.5	0.33	2.3

The authors apologize for this error and state that this does not change the scientific conclusions of the article in any way. The original article has been updated.

