# Supplementary material for: Assessing potency and binding kinetics of soluble adenylyl cyclase (sAC) inhibitors to maximize therapeutic potential
Source: Front Physiol. 2022 Sep 28;13:1013845. doi: 10.3389/fphys.2022.1013845 (PMC9554468; doi:10.3389/fphys.2022.1013845)
Supplement: Supplementary file 1 [file Image2.PDF]

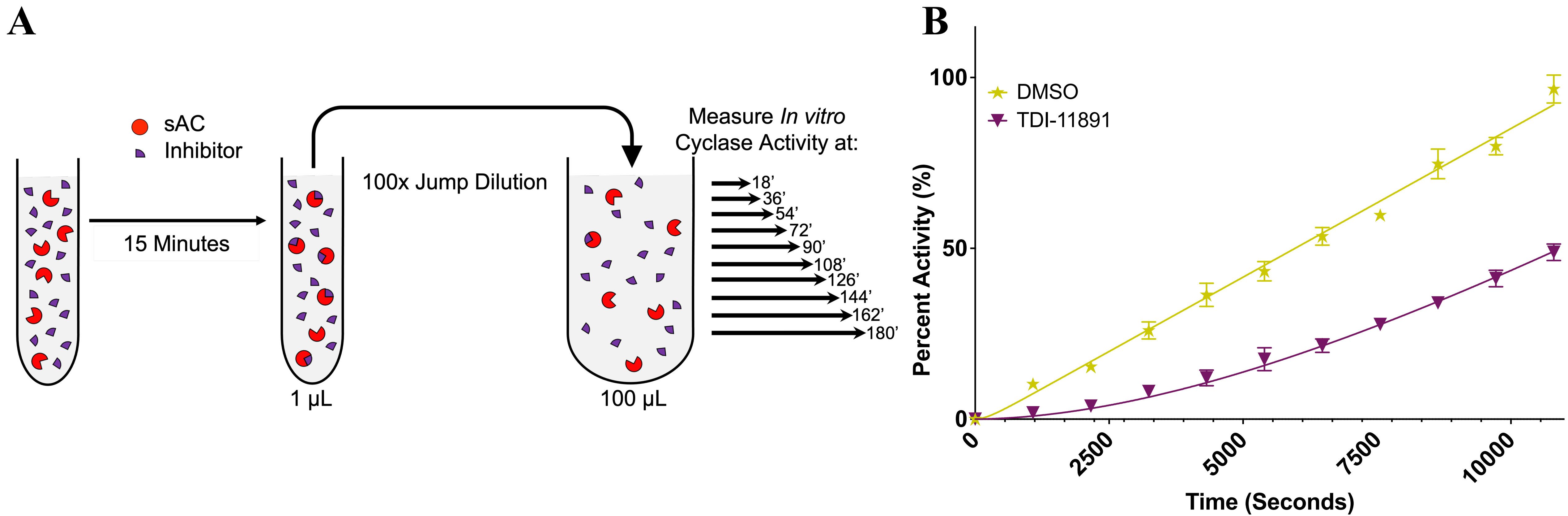

Supplementary Figure 2. **(A)** Schematic diagram of the extended jump dilution assay (figure adapted from BellBrook Labs "A Guide to Measuring Drug Target Residence Times with Biochemical Assays") **(B)** *In vitro* jump dilution curves of indicated inhibitors. All assays were done at 30°C in the presence of 2 mM ATP, 10 mM Mn^{2+} and ~0.25 nM of purified recombinant human sAC protein. sAC activity was measured every 18 minutes for 180 minutes. Data is normalized to respective DMSO-treated controls and is shown as mean ± SEM (n≥4).
