# Supplementary material for: Assessing potency and binding kinetics of soluble adenylyl cyclase (sAC) inhibitors to maximize therapeutic potential
Source: Front Physiol. 2022 Sep 28;13:1013845. doi: 10.3389/fphys.2022.1013845 (PMC9554468; doi:10.3389/fphys.2022.1013845)
Supplement: Supplementary file 2 [file Image1.PDF]

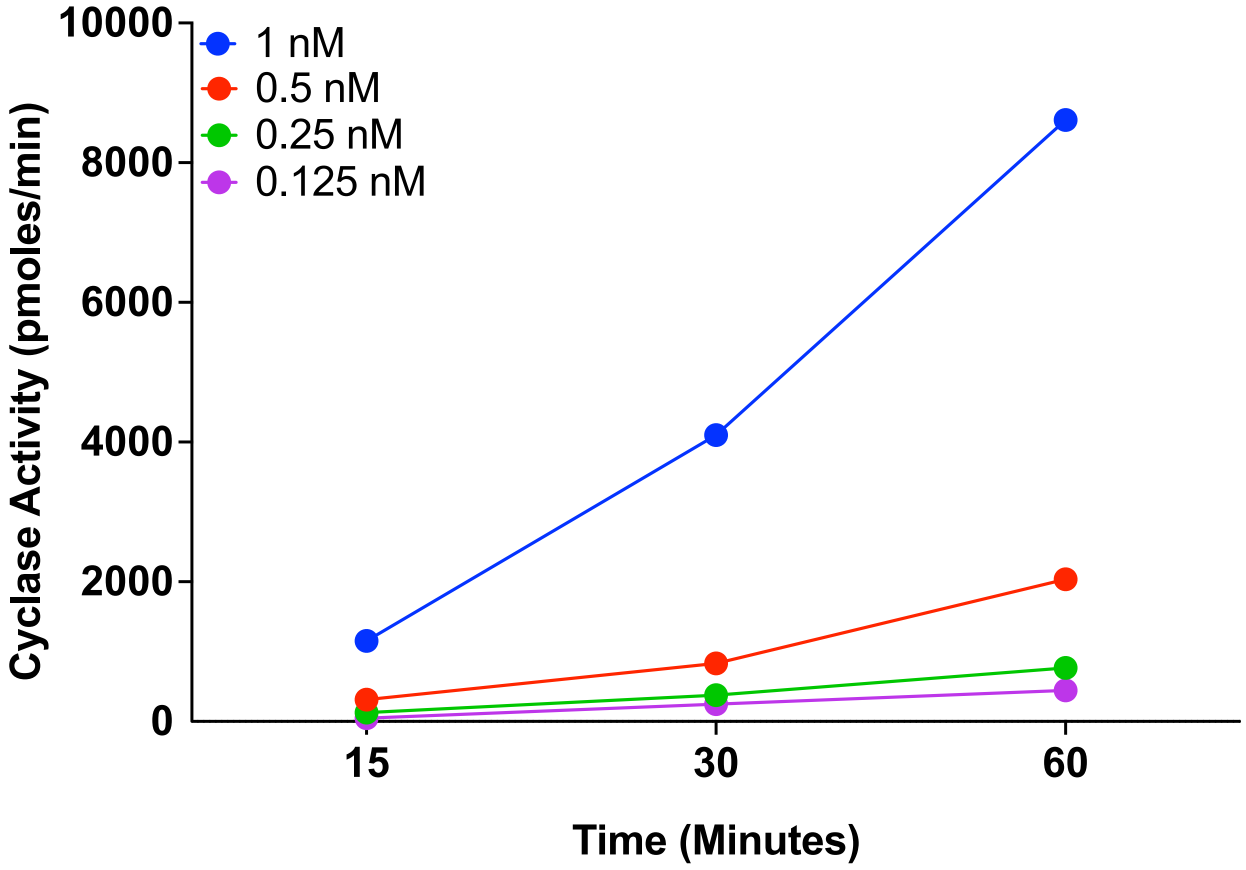

Supplementary Figure 1. Cyclase activity of various concentrations of sAC protein measured over a 60-minute period. Data was collected at 30°C in the presence of 1 mM ATP, 4 mM Mn^{2+}, and the indicated concentration of sAC protein.
